# Decoding the genomic symphony: unravelling brain disorders through data integration and machine learning

**DOI:** 10.1038/s41380-025-03330-4

**Published:** 2025-11-01

**Authors:** Matthew Bracher-Smith, Valentina Escott-Price

**Affiliations:** 1 UK Dementia Research Institute at Cardiff, Cardiff University, Cardiff, UK; 2 Division of Psychological Medicine and Clinical Neurosciences, School of Medicine, Cardiff University, Cardiff, UK

**Keywords:** Genetics, Biomarkers, Psychiatric disorders

## Abstract

Machine learning (ML) is revolutionising our ability to decode the complex genetic architectures of brain disorders. In this review we examine the strengths and limitations of ML methods, highlighting their applications in genetic prediction, patient stratification, and the modelling of genetic interactions. We explore how ML can augment polygenic risk scores (PRS) through advanced techniques and how integrating functional genomics and multimodal data can address challenges like rare variants and weak genetic effects. Additionally, we discuss the importance of embedding biological knowledge into ML models to enhance interpretability and uncover meaningful insights. With the ongoing expansion of phenotype-genotype datasets and advances in federated learning, ML is poised to compete with and surpass classical statistical methods in disease risk prediction and identifying genetically homogenous subgroups. By balancing the strengths and weaknesses of these approaches, we provide a roadmap for leveraging ML to unravel the genomic complexity of brain disorders and drive the next wave of discoveries.

## Background

Brain disorders are complex and often highly heritable traits that can be caused by a combination of genetic, physical, psychological and environmental factors [1–3]. Such complexity is evident in their diagnosis, which is often based on symptoms. There is no clinical biomarker for schizophrenia or other psychotic disorders: these conditions are usually diagnosed after assessment by a specialist in mental health, and only a postmortem brain biopsy can confirm the presence of a specific type of dementia [4]. Differentiation between brain disorders is further challenged by a pronounced overlap in symptoms and comorbidities [5]. Neurodegenerative disorders like dementia, for example, cause a range of psychiatric symptoms, including depression and anxiety, in addition to physical difficulties like incontinence [6]. The phenotypic complexity of brain disorders is mirrored in their genetics. This includes a broad range of genetic variation which impacts risk for psychiatric disorders [7], including common and rare variants, single nucleotide changes, small insertions and deletions, and large structural rearrangements such as copy number variations (CNVs) and trisomy 21 [8–12]. While disorders like schizophrenia are characterised by a wide spectrum of genetic variation including a high burden of rare variants [13], others may be characterised by common variants of stronger effect in genes such as *LRRK2* in Parkinson’s disease (PD), or *APOE* in Alzheimer’s disease (AD). This divergent genetic architecture magnifies difficulties in modelling; a single modelling approach is unlikely to work consistently across all brain disorders.

### The rise of additive models

Genome wide association studies (GWAS) have been the driving force behind cutting the Gordian knot. A focus on statistical power and simple models helped to push through early quagmires in candidate gene studies and onto the first robust genetic associations with brain disorders like schizophrenia [14]. Procedures for quality control and conducting GWAS are now routine and robust. Applying hundreds of thousands of simple univariable additive models with stringent thresholds for the strength of evidence of association has ultimately been instrumental in identifying the lion’s share of common variants associated with psychiatric disorders and neurological diseases [15, 16].

If GWAS has been the workhorse of association, then polygenic risk score (PRS) has carried the burden of prediction. PRSs were originally designed to summarise genome-wide genotype data into a single variable that measures genetic liability to a disorder or trait. PRS studies often reach sufficiently high statistical significance levels (small *p*-value) to suggest trait polygenicity, but prediction accuracy is usually not sufficient for clinical utility. For example, the predictive performance of PRS in schizophrenia, a highly heritable disorder, is an Area Under the Curve (AUC) of about 0.73, while in bipolar disorder the AUC is lower, at around 0.65 [17]. Nevertheless, PRS has been suggested as a useful tool for the selection of individuals for clinical trials in individuals of European ancestry across different traits [18–21]. Furthermore, the PRS prediction accuracy of some traits is relatively high. Accuracy for AD reaches 0.70-0.75, and even higher if the diagnosis is based upon pathological confirmation (AUC up to 0.84) rather than clinical assessment [22]. While the polygenic method undoubtedly introduces noise by including some variants that are not involved in disease susceptibility (i.e. false positives), this is more than offset by the increased power to identify those at highest or lowest risk of disease. The use of publicly available effect sizes from large GWAS, and the reduction to a single variable, also means the sample size requirements for adequate power in the test set and the multiple testing burden are relatively modest.

In addition to maximising power and interpretability, simple additive models lessen the computational burden and therefore cost of working with large datasets by being fast and using relatively little memory. Efficient implementations of statistical approaches for the biobank era have been a focus of recent years, with some approaches even forgoing generalised linear models in favour of linear approximations [23]. This trade-off means that more complex modelling approaches must also factor-in increased computational and cost needs.

### Shortcomings of simplicity

Addressing complexity in data with simplicity in modelling has been both computationally tractable and hugely successful in alleviating early concerns that associations may not be genuine. The strengths of GWAS and PRS lie in their ability to provide robust, reproducible insights into genetic associations under additive models. However, a growing awareness of their limitations in capturing genetic complexity has emerged. Risk is not only determined by the individual presence of factors, but also in how they combine. GWAS and PRS assume that independent variants combine additively, both for alleles within and across loci, to influence disease risk. This has been invaluable but increasingly stands in contrast to findings in statistical genetics and the biological intricacies of disease mechanisms.

For instance, sample sizes in GWAS have increased dramatically [24], yet they still fail to explain the level of heritability observed in twin studies for brain disorders [25]. GWAS-based heritability estimates also rely on the assumption of additive effects, which is equivalent to looking for only the main effects of common variants contributing to disease risk. In the genetics of complex diseases, it remains unclear whether, and to what extent, non-additive genetic interaction effects contribute to risk. In Alzheimer’s disease, evidence of the huge discrepancy in disease risk depending on *APOE* status, and the differential biological effects such as amyloid deposition and microglial activation make it likely that such interactions do exist. Apart from additivity, classical models also typically assume predictors are independent, but treating every predictor individually without jointly estimating the effects also ignores a central tenet in clinical prediction modelling - that the effects of a predictor should be estimated jointly with others [26].

Association testing with GWAS and genetic risk prediction modelling through PRS have become common approaches in identifying associations and for assessing an individual’s risk of developing a given disease. While these approaches have been effective, their foundations mostly stem from ideas in the early 20^th^ century [27], and were developed to address concerns around false positives in early linkage and association studies, and the computational limitations of the time. Over the last 15 years since the first schizophrenia GWAS and PRS, the field has transformed. There is now have a preponderance of heterogenous, complex data, unprecedented computational power, and an array of flexible modelling techniques. This convergence offers a pivotal opportunity to move beyond simplicity and begin untangling the intricate symphony of genetic risk. Previously, we have reported systematic reviews assessing predictive performance and risk of bias of machine learning (ML) in psychiatric disorders [28] and AD [29] using purely genetic data. Here we take a narrative approach to consider the broader context of brain diseases, multi-modal data integration and advances in learning methodologies which will likely underpin the next wave of advances.

## Decoding complexity with machine learning

ML is often divided into supervised, semi-supervised learning and unsupervised learning. Unsupervised learning has been applied extensively in genomics and other omics fields, particularly for dimensionality reduction using methods such as principal component analysis (PCA). These are used frequently to handle high-dimensional data and are useful for identifying subgroups where no labels are available. Here, we focus on supervised and semi-supervised methods. Supervised methods include a number of now well known techniques such as neural networks (NNs), support vector machines (SVMs), random forests (RFs), and gradient boosting machines (GBMs). These may be applied to classification tasks, for assigning discrete classes, or regression problems, for predicting a continuous outcome. Lastly, semi-supervised methods are suitable for scenarios where data are partially labelled, such as in large meta-analyses which pool together data where cohorts may lack a uniformly defined outcome or consist of unscreened population samples which are often assumed to be controls.

A common argument for using machine learning approaches is that the exact effects of a variant on a specific outcome, in a given population, are often unknown. Where half a million genotyped variants are available, it is impractical to pre-specify known models for each of these or thoroughly check if assumptions for a regression model are met in each case. Traditional methods for genetic prediction specify how variants affect traits a priori, often taking all effects to be additive by default. Unlike these, machine learning approaches seek to estimate some function that maps from predictors, such as genotypes, to an outcome, like disease status. As such they do not enforce a set relationship between variants themselves, or variants and the outcome.

However, researchers applying ML models should be aware that they are not completely free from assumptions. While they do not prespecify a genetic model, the heuristics and algorithmic frameworks used in learning implicitly define how types of genetic variation are handled. In training, search for approximations of the true function mapping genes to disease is not random, but drawn from a limited space of models defined by the learning algorithm. For example, tree-based gradient boosting iteratively builds decision trees on the output of the loss function from previous trees. In turn, each decision tree partitions the predictor space and calculates risk for the subgroups in its terminal nodes. For rare variants, in which only 0 or 1 copies of the risk allele are observed in training data, a decision tree will only split between 0 and 1, so that individuals with 1 or 2 risk alleles are treated the same in predictions. This incidentally learns a dominant model as a consequence of applying this specific algorithm to sparse data. In contrast, a linear regression would model the effect on y of a unit change in the number of risk alleles, enforcing an additive model. This illustrates that while ML models are flexible and may be hypothesis-free, they are not assumption-free.

### Applications in genetic prediction of complex traits

Making assumptions in learning is essential, however, as these guide the search for models and allow them to learn relationships between variants without the computational burden of examining every possible combination. This allows models to combat the curse of dimensionality, which is prominent in genetics and often makes an exhaustive search infeasible, through heuristic search and the blessing of non-uniformity [30]. Combined with rigorous procedures for model tuning, ML methods are able to balance detection of complex patterns with overfitting.

In practice, ML methods have been employed to make predictions from genotypes, with the potential to bring improved prediction of outcomes; however, their current performance is unclear [28, 29]. Based on systematic reviews by us and others, the performance of machine learning methods has been highly varied (0.48-0.95 AUC) and differed between schizophrenia (0.54-0.95 AUC), bipolar (0.48-0.65 AUC), autism (0.52-0.81 AUC) and anorexia (0.62-0.69 AUC) [28]. For Alzheimer’s disease risk prediction AUC results have also varied (0.49-0.97) [29]. Given that genetic prediction for complex traits is bounded by heritability and the disease prevalence [31], these results match and outperform the theoretical maximum prediction accuracy. For example, in AD using PRS, an AUC of 0.82 was achieved assuming single nucleotide polymorphism (SNP)-based heritability h^2^ = 0.24 and life-time disease prevalence of 2% [19]. Nevertheless, the reported high accuracy could also be a result of one or more biases, which stems from study design and analysis flaws: choices related to predictor selection, hyperparameter tuning, validation methodology, and test set exposure during training.

The ability of machine learning methods to predict schizophrenia or other psychiatric dis- orders from genetics remains unclear. Attributes of studies which elevated risk of bias for analysis often relate to information leaking from the test set to the training set. Furthermore, comparison between machine learning, logistic regression and polygenic risk scores is hampered by low effective sample size. These limitations can be dealt with adequately by considering simulations. Here, for any given population parameters, a large external sample can be simulated and used to inform hyperparameter choices separately from any training data, avoiding the possibility of information leaking. In addition, additivity of genetic effects, and deviations from this, can be investigated alongside polygenic risk scores with and without prior information.

Disease risk prediction so far using ML applied to genetics, as measured by AUC, is comparable to PRS [32, 33]. A recent genome-wide machine learning study on the largest European databank for Alzheimer’s disease [34] identified putatively novel loci but also found no predictive improvement beyond PRS. Several factors contribute to this. Firstly, SNPs generally only correlate with causal variants, which limits the detection of nonlinear effects and interactions—the primary advantages ML has over PRS. Secondly, genetic predictors are relatively weak compared to others (e.g., biomarkers [35]), leading to an upper bound for AUC in complex trait genetics that is significantly below 1 [31]. Weak predictor-response relationships pose inherent challenges for flexible models, and currently, complex models may lack sufficient power to improve AUC substantially. Thirdly, large GWAS identify SNPs with small association effect sizes in summary statistics, though these effects may be larger in more homogeneous samples. For instance, the odds ratio (OR) for *APOE* is approximately 3.4 in cohorts with a mean age of ~72-73 years [36] but decreases in samples over 90 years old [37]. In pathology confirmed samples, typically older than clinical cohorts, some GWAS-derived SNP effect sizes are larger than those reported in clinically-assessed AD GWAS [22]. Homogeneous datasets in terms of age, population, and cognitive scores (e.g., the Alzheimer’s Disease Neuroimaging Initiative (ADNI) [38]) tend to show higher PRS AUC than clinical samples [39, 40]. Thus, while large GWAS meta-analyses provide summary statistics enabling PRS to achieve moderate AUC across datasets, they lack the specificity required for high accuracy due to averaging effect sizes across studies with varying recruitment criteria, outcome definitions, and genetic ancestry.

### Unravelling genetic interactions

**Fig. 1:**
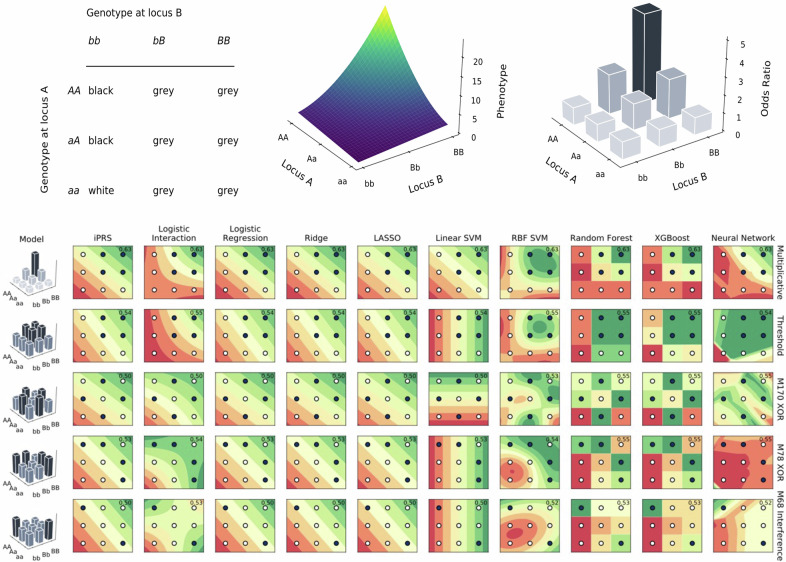
Handling epistasis in machine learning. Top: types of epistasis, as Bateson’s definition (left), a two-locus interaction on a quantitative trait (middle) and on the odds scale for a binary trait (right). Bottom: decision boundaries displayed by contour plots under simple 2-SNP interaction models when *theta* = 0.5 and minor allele frequency (MAF) = 0.5. X and Y axes indicate two loci, with dark points highlighting genotypes with increased risk. Effective classifiers should highlight dark points in green and white points in red. AUC, annotated on the top right of each subplot, does not use a single threshold, and so classifiers may have high AUC but still assign both light and dark points to the negative class. iPRS denotes an internal polygenic risk score which is trained from data in the training split, like all other models, rather than external summary statistics. Logistic interaction refers to a logistic regression model with main effects and an interaction term, i.e. logit(y) ~ ß_0_ + ß_1_SNP_1_ + ß_2_SNP_2_ + ß_3_SNP_1*_ SNP_2_. Five types of two-SNP interaction models, comprising multiplicative, threshold, two XOR and one interference model were used, denoted by their code assigned by Li and Reich [43].

For these reasons ML approaches have been explored widely for their ability to detect interactions [41, 42]. Such epistatic effects go well beyond Bateson’s two-locus masking effect (Fig. 1), including 512 models for two-locus fully-penetrant classification problems alone [43]. Random forests have been extensively explored for detecting genetic interactions, with modifications aimed at improving their ability to identify such effects [44–46]. They have been adapted for high-dimensional data [47] and applied to conditions like rheumatoid arthritis [48] and age-related macular degeneration [49]. Many studies have historically focused on variable importance measures (VIMs) or adaptations to screen for interactions [50, 51].

Gradient boosting has been less widely applied but has shown promise for identifying interacting SNPs in schizophrenia [52] and complex traits [53]. SVMs have been combined with multifactor dimensionality reduction [54] and applied in PD [55]. Neural networks have shown mixed performance in modelling interactions, sometimes outperforming traditional methods like logistic regression and RFs [56, 57].

Despite a large literature on ML and interactions, there are relatively few studies in which third parties have systematically compared what different methods can learn from interaction data (for example [58]). Given that each type of ML model makes different assumptions in learning suggests that, when estimating a decision boundary to separate to classes, they will not all learn exactly the same boundary. This is both intuitive and well established in the literature. An example illustrates this using simulations to gain a fundamental understanding of the behaviour of supervised ML approaches in the presence of main and interaction genetic effects [59]. This simulation study examines ML models trained on five distinct interaction types, representing diverse and contrasting scenarios (Fig. 1). From this example, which shows the decision boundaries from a single simulation, it is clear that ML methods are generally more precise than Logistic Regression (LR), but that this does not always translate into improved AUC. However, each specific ML method tends to perform best for specific interaction patterns. For example, the exclusive-or (XOR) pattern is learned best by RBF SVMs, which can be particularly flexible, while the “threshold” pattern is better detected by XGBoost, and a “multiplicative” pattern is sufficiently well captured by LR with an interaction term, based on Fisher’s definition of epistasis [60].

More generally, it is common to report on detection of interactions but much less common to report on predictions from interactions. This partly because replicating an interaction is particularly difficult: small sample sizes for genotype combinations, alongside differences in minor allele frequency (MAF), effect size and linkage disequilibrium (LD) across populations compound [61]. It is also because, whether using an approach which implicitly detects interactions, or one that explicitly searches for them, the impact on prediction accuracy is often minimal. Challenges like the need to aggregate rare variants or constrain the weak effects of common variants to learn effectively further amplify existing limitations. Model performance also tends to degrade under imperfect conditions, highlighting the limitations of using genetic data alone. Enriching genetic data with information from other modalities may enhance models by providing constraints and amplifying biological signals. However, multiple challenges remain in model development and validation if these improvements are to have an impact.

## Open challenges in applying ML

### Mitigating risk of bias

**Fig. 2:**
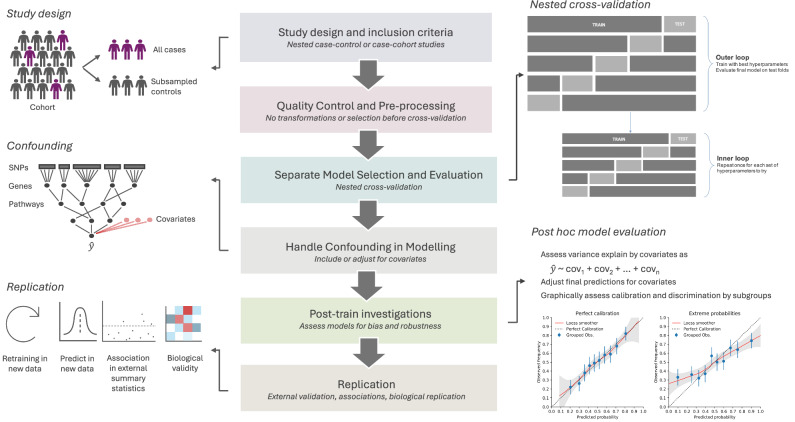
Building Better Models: Common Pitfalls of ML Applications in Brain Disorder Genetics.

Though novel and exciting applications continue to emerge, there are several clear challenges present across models which have been applied in brain disorders and beyond (Fig. 2). A number of these are specifically associated with the use of ML in genomics including overfitting in high dimensions, addressing data heterogeneity, and procedures around model selection and reporting [62]. Recent reviews [28, 29, 63] highlight that key steps in model development and validation are frequently either not performed or go unreported, sometimes leading to overstated conclusions. Such omissions raise questions around data leakage in training, which remains an important issue in ML study designs. Prior to modelling or data processing, studies may utilise a design that is sub-optimal for the target end point, such as a case-control design from which accurate probability estimates cannot be obtained. Nested case-control and case-cohort designs (Fig. 2) have been highlighted as potentially more efficient and representative [64, 65]. For example, a recent case-cohort approach was taken in the Danish national register to evaluate neural networks for cross-disorder risk prediction [66], and work from our group has employed a nested case-control design to compare ML approaches for prediction of schizophrenia in the UK Biobank [32]. These rely on subsampling a larger cohort, maintaining statistical power and reducing computation [64], while allowing for prediction estimates which can be scaled to proportions in the original cohort [67]. As large databanks of health records and population biobanks become more available, employing these designs is becoming more necessary.

Common sources of bias include transforming variables before cross-validation, and the absence of an independent test set [68]. Frequently, issues arise from a failure to separate the choice of an optimal model in training (model selection) from its final performance evaluation. When tuning hyperparameters, it is a common issue for researchers to use the same cross-validation rounds for both model selection and evaluation, leading to overly optimistic performance estimates. Nested cross-validation (Fig. 2) addresses this by separating the two processes, with an outer loop for evaluation and an inner loop for model selection, running as many times as there are parameter combinations [69, 70]. This approach provides a more accurate estimate of model error but is computationally intensive and under-utilised in genetics. While split-sample validation may suffice for extremely large datasets, the cost of acquiring medical datasets, and subsequent small sample size, often necessitates nested cross-validation. A systematic investigation of various data leakage factors is an underexplored topic in the genetics of complex traits and warrants further investigation.

### Confounders

Confounding is an ever-present issue in epidemiology [71]. The literature for handling it is extensive and varied in classical statistics [72, 73]. In ML, several such methods can be easily lifted-over from medical statistics. Prior to modelling, strict quality control procedures used in GWAS and PRS studies can similarly be applied in ML studies [74, 75]. However, some ML approaches remain difficult to adapt in the face of confounding. Neural networks, for example, can include covariates which only directly connect to the final layer, with predictions then made from all non-covariate connections to the output node. In a random forest, including covariates as predictors naturally integrates them into the decision trees alongside other variables, making it difficult to disentangle their effects from those of non-covariate predictors when making predictions or drawing inferences. As such, regressing covariates from both the predictors and the outcome before modelling is often used [76], but is a sub-optimal approach. More complex types of confounding, which cannot be accounted for by simply including covariates, such as collider bias, are not easily handled. Similar issues are present for genetic data, where techniques for handling genetic ancestry and diverse cohorts are not always easily implemented or applied.

Population structure is a significant source of bias in genetic analyses, affecting associations and predictions [77–79]. Supervised machine learning methods have been highlighted as easily able to learn such populations from labelled data [80], though performance of flexible models has also been reported as similar to linear approaches [81]. The degree to which bias from mishandling of population stratification in machine learning studies is unclear, as studies have mainly evaluated prediction of populations directly using supervised ML. While modifications to models or modelling procedures have been proposed [76, 82], their efficacy has not been robustly validated. Adoption of strategies in other fields which propose reporting the variance in model predictions explained by confounders and a systematic comparison of ML methods and the degree to which population effects are handled in prediction of brain disorders, would be valuable in mitigating risk of bias. Despite issues, lessons from causal inference have positively influenced applications in the biosciences, and efforts to improve debiasing or deconfounding in ML have grown [83–86]. Approaches discussed below, such as propensity score weighting which is often used to address confounding by indication [87], may be expanded to cohorts with genetic data to untangle effects in the presence such confounding. More broadly, poor study design and reporting in ML have been addressed by multiple groups. We point researchers to several articles [88, 89], in particular the TRIPOD + AI guidelines [90] which provides a checklist for improving reporting of artificial intelligence (AI) models in medicine.

### Replication of ML results

Concerns about whether signals are genuine or influenced by biases in the training data necessitate discussions about replication. In ML, replication can be interpreted in various ways (Fig. 2), including replicating the same effect of predictors in a new dataset through the same or other approaches, replication of effects across different approaches, or complete retraining of the model and demonstrating consistent predictor effects in an independent external dataset. The latter is often infeasible. In particular, cohorts for neurodegenerative diseases are affected by inclusion of controls for whom the outcome is unmeasured, or who are unlikely to have developed the disease yet, which differ in external datasets and so negatively impact likelihood of replication. These effects are exacerbated by the ability of flexible ML algorithms to identify complex patterns. They are consequently more likely to encounter similar issues in varying effects, outcome measurement or LD with causal variants, as noted for replicating interactions across datasets in general. These replication challenges highlight the importance of external validation. However, while external validation is a robust threshold for publication, it may inadvertently exclude valid patterns or signals. A focus on careful selection of cohorts for training, testing and replication is vital to ensure novel insights are carried forward. Replication across different ML approaches, by comparison, is not guaranteed or even expected. As highlighted for interactions, each algorithm may detect unique patterns in the data or classify individuals differently based on traits or symptoms [32].

### Ethical considerations and generalisability

Challenges in handling of population structure and replication in ML point toward a more general issue of generalisation, particularly across diverse populations. Though sample sizes in non-European genetic cohorts have increased, they are far from proportional to global population sizes [91], a disparity which limits applicability of models globally [92]. In psychiatry, where key predictors include genetic and social factors, there are genuine concerns around misuse, for instance if a predictive model for schizophrenia has a higher false positive rate for a minority ethnic group. While increasing diversity of the data is the ultimate goal, strategies are required to mitigate issues in the models built with the data available now. To achieve this, it is essential that AI-based interventions make algorithmic fairness a key priority through evaluation of model outputs to ensure performance is equitable across different groups. In addition, emerging methods in causal machine learning (discussed under “emerging opportunities”) offer a principled framework for training models which avoid spurious associations in data and reduce the risk of perpetuating societal biases [93]. However, such technical approaches must be combined with clear clinical guidelines for the responsible communication of AI-derived information to prevent patient stigmatisation [94]. More broadly, the FUTURE-AI framework offers guidelines to researchers looking to develop trustworthy AI applications in healthcare [95].

### Limited data and data access

Machine learning research relies heavily on large datasets to train models effectively and achieve accurate predictions [96]. The most frequent limitation of ML studies using genetics and other data modalities as predictors is sample size, with the total number of participants from case-control studies often numbering less than a thousand, whilst the number of predictors may comprise several thousands. For example, as of 2021 the majority (85%) of studies applying ML to predict Alzheimer’s disease from genetics alone used the publicly available Alzheimer’s Disease Neuroimaging Initiative (ADNI) dataset [29], demonstrating clear overreliance on a single data source of European origin. Conversely, population-based databanks like UK Biobank (www.ukbiobank.ac.uk) or All of Us (allofus.nih.gov), have a large sample size but are not sufficiently enriched for cases, as brain disorders have low prevalence in the general population and these cohorts often include younger individuals who are unlikely to have developed neurodegenerative disorders. ML studies must therefore push for larger sample sizes, necessitating the combination of data from potentially diverse sources, as in large meta-analyses from consortia. Access to and sharing of data is essential for achieving this.

Despite significant advancements in development of AI and ML for healthcare applications such as disease diagnosis, prognosis, therapy response prediction, survival estimation, and patient stratification, only a limited number of ML tools have successfully transitioned into clinical practice [95]. The data privacy regulations (e.g. Health Insurance Portability and Accountability Act (HIPAA), the European Union General Data Protection Regulation (GDPR)) mandate strict guidelines to protect individuals’ privacy, requiring explicit consent for data use and imposing constraints on data storage and sharing, and outline legal and financial penalties for non-compliance. The standard practice for securing biomedical and genetic data involves encrypting data at rest, employing a secure computing infrastructure, and deidentification strategies [97]. While they aim to balance data protection with technological progress, its impact on data accessibility remains a concern for researchers and organizations striving to develop innovative AI solutions [98].

## Emerging opportunities in machine learning

### Advances fuelling AI

Despite the challenges outlined above, ML and AI methodologies continue to advance rapidly and play a crucial role in uncovering complex patterns within high-dimensional data. Advances in biotechnology have enabled reliable recording of various aspects of human biology, such as genetic data and other commonly used biomarkers (e.g., cerebral blood flow and brain imaging). These advancements have led to the accumulation of large biological datasets that ML algorithms can analyse to classify participants or predict membership in predefined categories [99]. The combination of genetic data with other data modalities often leads to complexity, which cannot be processed easily by humans in an un-biased way [100].

### Improved interpretability with explainable AI (XAI)

While learning from this complexity has traditionally been difficult, efforts in explaining the resulting models are now well-developed. The interpretability of machine learning models has been significantly enhanced by the introduction of SHAP (SHapley Additive exPlanations) values [101, 102] and related approaches. Though alternatives exist and continue to be developed, SHAP provides a unified approach to understanding the contributions of individual features to a model’s predictions by offering a consistent and mathematically grounded method based on Shapley values from cooperative game theory. Despite widespread discussion of ML models as black boxes, researchers are now able to obtain detailed explanations of predictions at the global (averaged across individuals) and local (per-individual) level, cluster individuals by their predicted values from all or a selection of predictors, and explain how a prediction for a specific individual was derived. It is an under-appreciated benefit that explainable AI (XAI) approaches can offer greater insight at the individual level than effect sizes from a regression which show the change in the outcome for a given predictor averaged across all individuals.

### Causal machine learning

Approaches like SHAP are often applied under a traditional ML paradigm, where researchers aim to explain improved prediction of an outcome or identify novel risk factors. This primarily relies on training a model which maximises prediction and subsequently explaining the outputs. In contrast, causal machine learning explicitly aims to model causal effects rather than associations, an approach that has become increasingly important as large electronic health records (EHRs) have become more accessible to researchers [103]. This relies on a formal framework, where the causal structure of the problem is considered, often using a directed acyclic graph (DAG) [104]. In addition to careful design of the study and specification of causal relationships, key methodological steps include defining the causal quantity of interest, assessing underlying assumptions, selecting an appropriate ML model, and conducting robustness checks [105]. Handling confounding is at the core of causal ML, therefore addressing many of the concerns raised about open challenges from past efforts in the genetics of brain disorders. Furthermore, these approaches inherently focus on estimation of individual treatment effects (ITEs), rather than average treatment effects (ATEs), which support clinical decisions more directly.

While SHAP applied to standard ML helps explain how variable changes influence model predictions, it does not establish whether these changes correspond to actual causal effects in the studied individuals. By contrast, causal ML seeks to quantify the impact of interventions on outcomes and answer “what if” questions. This ultimately shares much of the framework, principles and techniques from causal inference in statistics, while leveraging the ability ML models to handle complex data generating processes. This may involve using ML for modelling treatment effects, or in other areas such as modelling the effects of covariates on the likelihood to be treated. However, applying causal learning remains difficult in practice. Researchers must confront the fundamental problem of causal inference – that the counterfactual is not observed – and address assumptions including the stable unit treatment variable assumption (SUTVA), positivity and ignorability. Methods for robust uncertainty quantification in causal ML are also still evolving [103], though implementations such as causal forests provide this [106]. In neurodegenerative diseases, causal ML approaches have already been applied to EHRs to identify drugs for repurposing in dementia, where a long short-term memory (LSTM) model [107] was used to estimate the longitudinal effects of covariates and mitigate indication bias [108]. We expect similar applications to become more popular, particularly when used in deep learning approaches which integrate multimodal data for modelling risk factors or confounders.

### Multimodal data

**Fig. 3:**
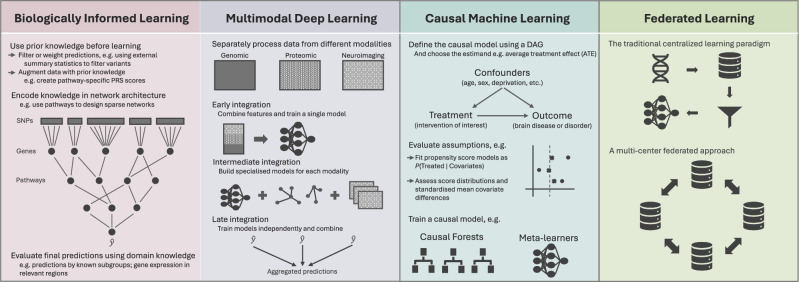
Expanding the ML toolkit - unravelling the complexity of brain disorders with causal, federated, multimodal and biologically informed learning.

Alongside progress in building interpretable models through careful design and analysis, models are also being enriched by inclusion of information from diverse sources (Fig. 3). Current diagnostic criteria for most brain disorders continue to rely heavily on clinical assessments, such as medical and family history, clinical interviews, and medication records. Over the past decade, significant advancements have been made in multimodal neuroimaging and genomic techniques. Moreover, blood and cerebrospinal fluid (CSF) biomarkers have been rapidly and successfully developed to identify individuals with prodromal dementia, particularly Alzheimer’s disease. However, no single measure can precisely define psychiatric or neurodegenerative disease. For instance, our research, along with that of others, shows that variations in CSF and plasma biomarkers are not fully explained by genetic factors but can significantly enhance disease risk prediction [109]. Furthermore, these biomarkers were found to be associated with age at the time of sample collection, suggesting sensitivity to age-related factors or preclinical neurodegenerative pathologies. Given the current state of knowledge, it is unrealistic to expect any single measure to adequately assess complex brain functions.

Decades of traditional neuroscientific research aimed at identifying structural and functional brain differences associated with major brain disorders have largely relied on multivariate statistics and relatively simplistic brain models. To date, these approaches have proven inadequate in uncovering the underlying causes of such disorders and in enabling reliable, individualised diagnoses [110]. In recent years, numerous studies have applied ML techniques to structural magnetic resonance imaging (sMRI), functional MRI (fMRI), genetic data, and selective phenotypic or clinical data to diagnose brain disorders. These studies aimed to leverage multimodal data to investigate the mechanisms and pathways involved in the development and progression of dementia (for example, AD and PD [111], and progression [112]), schizophrenia [113], depression [114], and autism spectrum disorder [115]. However, there remains no consensus on the appropriate ML methodologies, predictor sets, or hyperparameter choices.

Both deep learning and multiple kernel learning (MKL) have received continued attention. MKL requires the use of multiple kernels such that data from different modalities each use a distinct kernel. The method combines these to form a meta-kernel and derive similarity scores for samples in different data sources, ultimately feeding into a classification approach like SVMs. These have been used to combine imaging, proteomic and genetic data in Alzheimer’s disease, for example [116], with general approaches compared recently [117].

Though MKL is used for specific instances of data integration, deep learning has emerged as the leading approach for fusion of diverse data modalities due to its flexibility and the possibility of creating end-to-end workflows with less requirement for feature engineering. A distinction between early, intermediate and late integration remains prominent in the field, though intermediate integration is often highlighted as still being able to exploit distinct attributes of data types, unlike early integration, while also capturing interactions between modalities, as opposed to a late integration approach [118].

Related to this is the topic of applying feature selection (FS) when incorporating data from multiple modalities. Though modality-specific filters may be required as part of quality control, predictors should ideally be considered together during FS to ensure any interaction between them is accounted for. However, genomic data alone are particularly “wide” and combining them with other omics predictors necessitates some attempt to constrain dimensions, both to reduce computation and improve generalisation. Wrapper and embedded FS on the full combined data consider predictors together, but require significantly more computational resources. This and a drive for simplicity often mean researchers focus on pre-filtering features before modelling. In doing so, researchers should be aware of the trade-off made by pre-filtering on main effects or performing a modality-specific screen, such as taking only independent SNPs below a certain *p*-value threshold. Such an approach may prove computationally necessary, and a careful approach can effectively reduce dimensions while maintaining core signals likely to interact. However, it may also remove features which combine non-additively or are important only in the context of data from another modality. Early integration is less susceptible to this issue where FS is performed on the concatenated data, as is intermediate integration where interactions primarily occur between emergent features at later points in the network. Combined feature selection approaches, such as joint estimation of effects in a penalised model, or cross-modal attention may help to apply FS without information loss which is essential to inter-modality interactions.

A variety of architectures are in use for data integration. Extensive incorporation of imaging modalities has meant convolutional neural networks (CNNs) remain popular, e.g. [119], which typically make use of a late fusion approach for combining imaging data [118]. Applications also include recurrent neural networks (RNNs) for longitudinal data in EHRs [120], graph neural networks [121], and more recent use of generative approaches like variational autoencoders (VAEs) for handling missing data like DeepIMV [122] and GLUE [123]. A use of conditional restricted Boltzman machines (cRBMs) as part of the PsychENCODE Consortium is also noteworthy for its scope, range of genomic, transcriptomic and epigenomic resources, and freely available model weights [124]. Models employing a late integration strategy may only apply deep learning for a specific modality, often neuroimaging, and combine the final outputs in tree-based ensemble methods, for example [125, 126].

In addition to integrating data from multiple modalities, biological knowledge can also be encoded directly into deep learning architectures directly through biologically interpretable neural networks [127] (Fig. 3). These seek to define layer connections or weights through prior knowledge, such as hierarchical gene ontology data or regulatory relationships [128]. The term is sometimes used expansively to cover both knowledge-guided deep learning architectures and multi-modal data integration [129]. More recently, biologically informed network architectures have been combined with multi-modal inputs to enhance genetic prediction and model interpretability by incorporating expression quantitative trait loci (eQTLs) and gene regulatory networks in brain disorders [130], and by integrating methylation data, KEGG pathways and gene expression data in prediction of demographic and biomarker variables [128]. With wide usage of smartphones and wearables, digital data can also be easily collected and utilised for detection of a disease at early stages. For example, ML models trained using accelerometry data achieved better test performance in distinguishing both clinically diagnosed PD and prodromal PD up to 7 years pre-diagnosis [131].

### Emerging strengths of a federated approach

Despite the richness of diverse multimodal data and its importance in understanding the basis and cause of the disease, the inequality in resource of the owners, especially genetic data, has led to concerns of knowledge colonialism whereby data is taken but knowledge is not returned. Data privacy regulations also restrict or delay the access to human data even within a single country. Federated learning (FL) is a novel approach to address this (Fig. 3), wherein separate ML models, often neural networks, collaboratively train across diversely located and privately held data in situ, respecting ownership rights and privacy concerns. This contrasts with the classical central learning paradigm, and ensures only model parameters, and not data, are securely shared across sites with standard encryption procedures during weight updates. The application of FL to national and international data to assess and derive measures of disease risk therefore provides a means to both respect the rights of data holders while increasing the utility of disease risk prediction amongst diverse populations. This offers a promising solution to overcome the constraints raised by limited access to high-quality datasets.

Efforts to apply FL in medical data [132] and genetics [133] have already paved the way for further advancements. Recent work has also implemented a federated GWAS in age-related macular degeneration (AMD) and cancer data [134]. Future research has the potential to address common challenges such as heterogeneity across datasets. For instance, in genetic risk prediction, variations in allele frequencies or effect size distributions across cohorts can shift predictor distributions, potentially introducing bias. Federated PCA offers a strategy to identify outlying cohorts, which may benefit from tailored approaches such as subsampling to handle non-independent and identically distributed (non-IID) data during training [135]. Evaluating strategies for collaborative learning, including adaptive aggregation techniques or the sequential integration of cohorts, can help minimize bias and enhance the extraction of genuine biological signals. Additional incorporation of methods like weak supervision, a form of semi-supervised learning which can improve learning from unlabelled data, or multi-task learning (MTL), in which multiple output labels are used in a neural network, can further expand such federated approaches even to siloed datasets with missing outcomes or proxy measures.

For successful implementation of FL in health care, clear, widely accepted guidelines are required on how healthcare AI tools should be designed, developed, evaluated. These tools need to be technically robust, clinically safe, ethically sound, and legally compliant [95]. In parallel, privacy-enhancing technologies to safeguard the data are appearing, with a promise to broaden FL usage by providing means to share and analyse sensitive data while protecting privacy [97].

### The road ahead

One reason for the diagnostic delay of brain disorders is the increasing number of evaluations requested, which increases the waiting time for families to meet with a specialist. Developing innovative AI-based technologies will help overcome these issues and augment various diagnostic aspects in mental health care. The success of ML predominantly depends on the quality of data, features in the data, the choice of objective or loss function, and the selection of an appropriate model architecture and hyperparameters that best fit the research question. Although, studies aiming for the discovery of novel diagnostic biomarkers for brain disorders have been advancing throughout the recent years, the application of ML tools using genomics and neuroimaging data in brain disorders is still in its infancy.

As the number of AI models grows, the future will undoubtedly involve more interest in bringing these to clinical settings. Here ML models have the potential to bring important benefits by estimating individual treatment effects through causal ML or understanding how variables affect a specific prediction using explainable AI, both of which go beyond typical estimates of the average effect in the study population. This should be a source of great optimism. In practice, however, models are often mired in poor development, validation or reporting practices [136, 137]. While AI models in brain disorder genetics have drawn from areas such as computer science, genetics and neurology, efforts to bring successful models to the clinic will also need expertise from clinical prediction modelling [26]. This field is distinct from AI and ML, with established best practices that address several of the limitations in basic research, such as optimism bias (poor generalisation) and the need for external validation [138]. Additionally, it emphasises key areas like clinical utility and decision-curve analysis (DCA) [139]. A recent study on AI-improved prediction of atrial fibrillation exemplifies the unification of these fields [140]. The authors utilise expertise in deep learning and best practices in clinical prediction modelling by combining electrocardiogram data and PRS and demonstrating higher net benefit of the combined AI model through DCA. Prospective randomised controlled trials (RCTs), the gold standard for assessing the efficacy of an intervention, are relatively uncommon for AI. Trials often focus on diagnostic aids or decision support for clinicians, or chatbots for therapy-based interventions. To this end a recent RCT demonstrated improved clinical outcomes for LLM assistance in diagnosis of complex cases [141].

Beyond methodological rigor, an important challenge in clinical translation is ensuring that the studied population aligns with the target clinical population. Without this, even a well-validated model may perform poorly when deployed in practice. Model sharing and predictions are also import practical considerations. A notable benefit of traditional regression modelling is that the linear predictor can be easily shared, allowing exact variable weights in a risk model to be transparently reported and interpreted in publications. This enables clinicians to calculate risk scores for individual patients to identify those at elevated risk e.g. for clinical trials or screening programs for targeted prevention or early intervention. By contrast, ML models present challenges in sharing and implementation, as risk scores cannot be directly computed without access to the trained model. Deploying ML-based risk models requires storing the trained model (through serialisation techniques like pickling) and serving it in a production environment, technical requirements that demand specialised expertise beyond model development. A higher demand for resources in training and a need to deploy live models for prediction also adds a much greater financial cost to AI models.

Before the clinic, the path ahead for research will likely involve further uses of large language models (LLMs), which have had substantial impact on a broad array of areas. LLMs have been proposed for a number of tasks in bioinformatics [142], including feature selection and engineering in genetics [143], and highlighting functional gene convergence and gene prioritisation after analysis [144]. We expect use of LLMs for brain disorders and other areas to increase, particularly with use of a foundation model and retrieval-augmented generation (RAG) on specific bioinformatics databases.

### Future perspectives

AI technology is still relatively new in the field of risk prediction for brain disorders, and significant advancements are needed to develop more efficient and accurate predictive models. The inherent heterogeneity of brain disorders, coupled with simultaneous functional and anatomical changes, presents challenges for diagnosis and risk prediction. However, data and algorithms have now reached a threshold where ML can rival classical methods. Emerging approaches, such as federated learning, provide opportunities to move beyond traditional meta-analyses by integrating AI-based algorithms to harness the full potential of diverse datasets. Future efforts should focus on developing integrated methods or multimodal architectures that combine features from high-dimensional data to amplify biological signals and guide more effective model training. In genetic risk prediction, it is both necessary and feasible to identify genetically-defined clusters of individuals with distinct or overlapping pathologies, paving the way for more personalized and biologically-informed insights into brain disorders.

## Conclusions

The ability to condense and reduce large-scale data, effectively distinguishing signal from noise, while capturing the complexity of brain disorders makes data-driven techniques powerful tools for generating and validating hypotheses. Despite persistent challenges with bias and inadequate reporting that hinder clear progress, advances such as federated learning present exciting opportunities to incorporate more diverse data and deepen our understanding of brain disorders. Both large-scale approaches in data integration from different modalities with deep learning models, as well as more subtle uses of ML in augmenting PRS or existing linear models, promise to aid in unravelling the genetic components of these disorders. However, the future success of such endeavours depends on the willingness of researchers from non-computational disciplines to openly collaborate with mathematicians and computer scientists, their readiness to make data accessible, and a collective effort to carefully develop, interpret and report results. Ultimately, embracing these approaches has the potential to illuminate the underlying mechanisms of brain disorders, driving meaningful progress in research and clinical care.

## Data Availability

This review article is based entirely on publicly available data and literature. All data supporting the findings and discussions presented in this manuscript have been cited within the text and listed in the references. No new datasets were generated or analysed specifically for this study.
